# Severe *Plasmodium vivax* Malaria, Brazilian Amazon

**DOI:** 10.3201/eid1610.100685

**Published:** 2010-10

**Authors:** Márcia A. Alexandre, Cynthia O. Ferreira, André M. Siqueira, Belisa L. Magalhães, Maria Paula G. Mourão, Marcus V. Lacerda, Maria das Graças C. Alecrim

**Affiliations:** Author affiliations: Fundação de Medicina Tropical do Amazonas, Manaus, Brazil (M.A. Alexandre, C.O. Ferreira, M.P.G. Mourão, M.V. Lacerda);; Universidade do Estado do Amazonas, Manaus (M.A. Alexandre, A.M. Siqueira, B.L. Magalhães, M.P.G. Mourão, M.V. Lacerda, M.G.C. Alecrim);; Centro Universitário Nilton Lins, Manaus (M.A. Alexandre, A.M. Siqueira, M.P.G. Mourão, M.V. Lacerda, M.G.C. Alecrim)

**Keywords:** Malaria, Plasmodium vivax, chloroquine, primaquine, parasites, Brazilian Amazon, dispatch

## Abstract

We describe a case series of 17 patients hospitalized in Manaus (western Brazilian Amazon) with PCR-confirmed *Plasmodium vivax* infection who were treated with chloroquine and primaquine. The major complications were jaundice and severe anemia. No in vivo chloroquine resistance was detected. These data help characterize the clinical profile of severe *P. vivax* malaria in Latin America.

During 2000–2007, in Latin America, a total of 7,554,993 cases of malaria were recorded; 5,507,167 (72.9%) of these cases were caused by *Plasmodium vivax* parasites*.* Of the *P. vivax* malaria cases, 3,833,477 were reported in Brazil, mainly in the Amazon Region (*1*). Official data from the Brazilian Ministry of Health identify Manaus as one of the leading cities in terms of number of *P. vivax* malaria cases in Latin America (*2*).

Manaus (population 1,738,641 in 2009), the capital of the state of Amazonas in the western Brazilian Amazon, is clearly part of a new frontier in the economic development of the Amazon. In 2009, a total of 19,698 cases of malaria were reported in Manaus (annual parasitary index 11.3/1,000 population; 92.6% caused by *P. vivax*). Since the mid-1990s, as *P. vivax* was becoming the predominant malaria species in Brazil (*3*), severe cases and even deaths attributable to *P. vivax* infection have been reported anecdotally (*3*). A concomitant trend of increased hospitalization of *P. vivax–*infected patients was seen in a Manaus tertiary care center (*4*).

In 2000, one of the authors noted the increased clinical severity of *P. vivax* cases seen in this same hospital; the frequency of hospitalization was very similar to that of patients infected with *P. falciparum* (M.G.C.A., unpub. data). Other reports from the same reference center in Manaus have been published regarding unusual complications of *P. vivax* infection, such as severe rhabdomyolysis (*5*) and immune thrombocytopenic purpura (*6*). At the same time, a cascade of reports from areas where *P. vivax* malaria is highly endemic confirmed the clinical severity of the infections (*7*). However, to date, data are lacking on the distribution of severe *P. vivax* malaria, the relationship of patient age, and the identification of possible risk factors. This study describes the clinical features of *P. vivax* malaria in a case series of patients who were hospitalized in a tertiary care unit in the Brazilian Amazon and their clinical response to treatment with chloroquine.

## The Study

The Tropical Medicine Foundation of Amazonas is a tertiary care center for infectious diseases in Manaus (3°8′S, 60°1′W). In 2001 and 2002, a total of 13,056 cases of malaria were diagnosed in this institution (11,251 *P. vivax*), representing 65.1% of the total cases from Manaus. During the same period, 358 (3.2%) patients with *P. vivax* malaria were hospitalized. A retrospective analysis was performed of case-patients who fulfilled the malaria severity criteria of the World Health Organization (WHO) (*8*). These patients had an exclusive diagnosis of *P. vivax* malaria by thick blood smear (reviewed 2 times by experienced microscopists) and PCR, according to the technique described elsewhere (*9*).

PCR was performed on whole blood from all patients with *P. vivax* malaria, confirmed by microscopy and any *P. falciparum* severity criterion recommended by WHO. Blood specimens were routinely stored by the laboratory of the institution at –70°C. Full clinical information was available from the patients’ charts, and serologic tests for dengue virus, *Leptospira* spp., and hepatitis A, B, and C viruses were performed on available serum samples stored at –20°C.

Each patient was monitored for 28 days in outpatient clinics after beginning antimalarial treatment. Patients were routinely discharged only after parasitologic clearance and clinical recovery. Until 2006, chloroquine was still prescribed for patients with severe cases at a dose of 10 mg/kg on the first day and 7.5 mg/kg on the second and third days, followed by primaquine (0.5 mg/kg/day for 7 days), according to the Brazilian Ministry of Health guidelines. In 2006, WHO formally recommended the treatment of severe vivax malaria to be the same as that for severe falciparum malaria, because of the risk for an unrecognized mixed infection (*8*).

Seventeen patients were included in the analysis, and their clinical and laboratory data are shown in Table 1 and Table 2, respectively. All patients received chloroquine (orally or through a nasogastric tube) and primaquine. Acute respiratory distress syndrome (ARDS) (diffuse interstitial and alveolar infiltrate by chest radiograph and partial O_2_ pressure 40 mm Hg by arterial gas analysis) developed in patient 11 two days after she received chloroquine, and she died 3 days later. This patient had a negative thick blood smear from day 3 of treatment with choloroquine. The other 16 patients were followed up after discharge until day 28. None had clinical symptoms of malaria, and all thick blood smears were negative at days 7, 14, and 28.

**Table 1:** Clinical characteristics of 17 hospitalized patients who had parasitologic and molecular diagnosis of *Plasmodium vivax* infection, Manaus, Brazil, 2001–2002*

Patient no.	Year	Age/sex	WHO severity criterion	Duration of disease, d	Antimicrobial drug use	Erythrocyte transfusion	Concurrent condition	ICU	Death
1	2001	2 y/F	Severe anemia†	4	No	Yes	–	No	No
2	2001	9 mo/M	Severe anemia†	10	No	Yes	–	No	No
3	2001	3 y/F	Jaundice‡	3	No	No	HAV	No	No
4	2001	60 y/F	Acute renal failure§	7	No	No	Arterial hypertension	No	No
5	2001	1 mo/M	Severe anemia†	3	No	Yes	–	No	No
6	2001	40 y/M	Jaundice‡	9	No	No	–	No	No
7	2001	80 y/M	Acute renal failure§	6	No	No	–	No	No
8	2002	7 y/M	Hemoglobinuria/ jaundice‡	4	No	Yes	–	No	No
9	2002	5 mo/M	Severe anemia†/ ARDS	3	No	Yes	–	No	No
10	2002	28 d/M	Jaundice‡	5	No	No	–	No	No
11	2002	46 y/F	Severe anemia†/ ARDS	5	No	Yes	–	Yes	Yes
12	2002	48 y/F	Jaundice‡	7	No	No	–	No	No
13	2002	58 y/M	Jaundice‡	12	No	No	Diabetes	No	No
14	2002	47 y/F	Jaundice‡	8	No	No	–	No	No
15	2002	43 y/F	Shock¶/jaundice	4	Yes	Yes	–	Yes	No
16	2002	34 y/M	Jaundice	10	No	No	–	No	No
17	2002	50 y/M	Jaundice	7	No	No	–	No	No

*WHO, World Health Organization; ICU, intensive care unit; HAV, hepatitis A virus; ARDS, acute respiratory distress syndrome (tachypnea, shortness of breath, and signs of hypoxemia). †Hemoglobin <7 g/dL in adults and <5 g/dL in children. ‡Total bilirubin >3.0 mg/dL. §Creatinine >3.0 mg/dL. ¶Systolic arterial tension <80 mm Hg despite fluid therapy.

**Table 2:** Laboratory characteristics of 17 hospitalized patients who had parasitologic and molecular diagnosis of *Plasmodium vivax* infection, Manaus, Brazil, 2001–2002*

Patient no.	No. asexual parasites/mm^3^	Hemoglobin, g/dL	Total leukocyte count, cells/mm^3^	Thrombocytes, cells/mm^3^	Serum creatinine, mg/dL	Serum bilirubin, total/conjugated, mg/dL	Serum AST, IU/L	Serum ALT, IU/L
1	7,566	3.6	9,700	97,000	0.5	1.1/0.4	50	30
2	28,847	4.5	9,100	30,000	0.6	1.3/0.5	40	34
3	1,100	9.5	5,500	143,000	0.4	6.6/5.6	760	1,233
4	21,406	14.6	13,900	33,000	3.0	2.58/1.16	113	108
5	1,862	3.1	19,000	99,000	0.7	1.1/0.8	23	56
6	1,206	11.5	6,700	27,000	1.2	13.8/10.6	76	60
7	2,695	12.4	4,900	76,000	3.7	1.3/0.5	33	33
8	2,055	3.8	13,700	106,000	0.6	4.3/0.73	190	37
9	3,844	4.5	6,200	171,000	0.3	1.36/0.21	58	25
10	680	9.6	13,600	275,000	0.1	6.4/4.8	72	50
11	5,452	6.5	4,700	107,000	1.7	2.5/1.6	57	56
12	5,047	11.5	4,900	35,000	0.9	6.3/5.1	49	34
13	11,954	9.4	8,600	48,000	1.0	5.4/5.2	45	43
14	9,360	12.0	5,200	27,000	1.2	9.2/7.1	57	53
15	4,524	8.8	5,800	29,000	1.0	5.6/4.8	33	30
16	5,160	10.7	6,300	36,000	2.3	7.8/6.5	35	70
17	24,550	10.3	5,000	24,000	1.4	7.1/4.6	39	55

*WHO, World Health Organization; AST, aspartate aminotransferase; ALT, alanine aminotransferase.

The patients in whom complications developed exhibited a remarkably wide age range (28 days–80 years). This age range is similar to that seen in other case series from Latin America, such as in hospitalized children from Venezuela with severe anemia that required blood transfusions (*10*) and in adults from Rondônia (a state in the western Brazilian Amazon) who had severe anemia, jaundice, acute renal failure, ARDS, and shock (*11*). *P. vivax* malaria with ARDS has been reported in travelers who acquired the infection in Manaus (*12**,**13*). The finding of severe anemia in 4 of 7 children highlights the relevance of this complication in *P. vivax* infection (Figure), as shown in a prospective study from Papua, Indonesia (*14*). Nine patients sought treatment for cholestatic jaundice; for 8, jaundice was the only complication. The mechanisms involved are unknown.

**Figure:**
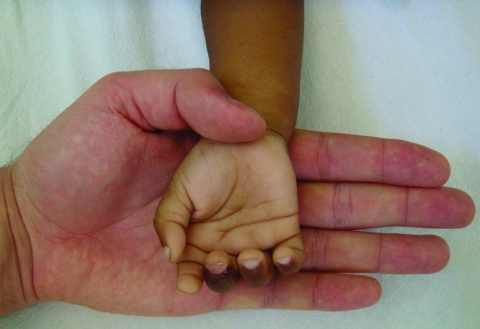
Hand of a 2-year-old child (patient no. 1) with severe anemia (hemoglobin level 3.6 g/dL), showing intense pallor, compared with the hand of a healthy physician. Photograph provided by authors.

The concomitant diagnosis of hepatitis A virus infection in patient 3 (Table 1) indicates that other infectious diseases should be excluded when characterizing severe *P. vivax* malaria. Also of note is the presence of thrombocytopenia in 15/17 patients (none had clinical bleeding), which suggests that this hematologic complication may be a surrogate marker of severity.

## Conclusions

The wide range of parasitemia found in our patients does not enable us to comment on the value of this variable as a determinant of severity. Sixteen patients recovered without the use of antimicrobial drugs; therefore, it is highly improbable that bacterial sepsis was a factor for severity in our case series. In unstable transmission areas (<0.1 autochthonous case per 1,000 persons per year), malaria in older patients may pose an additional problem because chronic diseases (e.g., arterial hypertension and diabetes) may predispose a patient to clinical decompensation.

In vivo chloroquine resistance was not detected in any of the cases that were followed up, despite recent confirmation of the phenomenon in this same locality (*15*). Because a reliable molecular marker of chloroquine resistance is lacking and parenteral artemisinin derivatives are recommended for treatment of patients with severe *P. vivax* malaria, studies that assess clinical severity and chloroquine resistance would be unethical. However, our findings suggest that chloroquine resistance would be a problem for individual patients and that the determinants of this resistance need to be clarified. Clearly, areas with chloroquine-resistant *P. vivax* also report severe *P. vivax* malaria, but we believe that these studies are not able to establish any firm causality. The finding of both phenomena in some areas may simply reflect high transmission of this species.

Our retrospective review illustrates the spectrum of severe *P. vivax* malaria in Manaus, and these results parallel the increasing clinical severity described in malaria-endemic areas such as Papua (Indonesia) and India. These severe *P. vivax* cases contribute to increased public health costs because of increased hospitalization and the need for intensive care and blood transfusions. The major complications in patients who required hospitalization were jaundice and severe anemia, although whether these complications were responsible for deaths is undetermined.

No clear severity criteria exist for *P.* vivax malaria. However, WHO criteria formerly defined for *P. falciparum* malaria seem to be applicable to most of the severe *P. vivax* malaria cases reported in hospital-based studies in the literature. PCR should be performed to rule out mixed infections and other common infectious diseases so that reports from different parts of the world are comparable. Despite the small number of patients, our data corroborate previous findings of severe disease found in areas where chloroquine-resistant *P. vivax* is being reported but suggest that establishing direct causality is not straightforward. We urgently need to know which clinical complications in *P. vivax* malaria are associated with death to validate severity criteria. A valid biomarker for chloroquine resistance would also enable associative studies to determine the association between resistance and severity.
